# Ultra-Capacitive Carbon Neural Probe Allows Simultaneous Long-Term Electrical Stimulations and High-Resolution Neurotransmitter Detection

**DOI:** 10.1038/s41598-018-25198-x

**Published:** 2018-05-03

**Authors:** Surabhi Nimbalkar, Elisa Castagnola, Arvind Balasubramani, Alice Scarpellini, Soshi Samejima, Abed Khorasani, Adrien Boissenin, Sanitta Thongpang, Chet Moritz, Sam Kassegne

**Affiliations:** 1 MEMS Research Lab, Department of Mechanical Engineering College of Engineering, 5500 Campanile Drive, San Diego State University, San Diego, CA 92182 USA; 2 Department of Nanochemistry, Istituto Italiano di Tecnologia, Via Morego 30, 16163 Genoa, Italy; 3 University of Washington, Division of Physical Therapy Departments of Rehabilitation Medicine and Physiology and Biophysics, Seattle, WA USA; 4 NSF-ERC Center for Sensorimotor Neural Engineering (CSNE), Seattle, WA USA

## Abstract

We present a new class of carbon-based neural probes that consist of homogeneous glassy carbon (GC) microelectrodes, interconnects and bump pads. These electrodes have purely capacitive behavior with exceptionally high charge storage capacity (CSC) and are capable of sustaining more than 3.5 billion cycles of bi-phasic pulses at charge density of 0.25 mC/cm^2^. These probes enable both high SNR (>16) electrical signal recording and remarkably high-resolution real-time neurotransmitter detection, on the same platform. Leveraging a new 2-step, double-sided pattern transfer method for GC structures, these probes allow extended long-term electrical stimulation with no electrode material corrosion. Cross-section characterization through FIB and SEM imaging demonstrate strong attachment enabled by hydroxyl and carbonyl covalent bonds between GC microstructures and top insulating and bottom substrate layers. Extensive *in-vivo* and *in-vitro* tests confirmed: (i) high SNR (>16) recordings, (ii) highest reported CSC for non-coated neural probe (61.4 ± 6.9 mC/cm^2^), (iii) high-resolution dopamine detection (10 nM level - one of the lowest reported so far), (iv) recording of both electrical and electrochemical signals, and (v) no failure after 3.5 billion cycles of pulses. Therefore, these probes offer a compelling multi-modal platform for long-term applications of neural probe technology in both experimental and clinical neuroscience.

## Introduction

Carbon is increasingly finding wider acceptance in micro and nanofabrication of a variety of sensors, actuators, microelectrodes, wires, batteries, fuel cells, thin-films, and neural interfaces^1–6^. Its unique tunable mechanical and electronic properties enabled by the availability of a range of possible hybridized bonds (*sp*^2^ and *sp*^3^) make it a versatile material. Further, carbon has a potential to assume even more far-reaching importance in applications varying from sensors to energy conversion and storage with discovery of newer carbon allotropes such as graphene (excellent conductivity and strength)^7^, Q-carbon (excellent hardness)^8^ and compressed glassy carbon (excellent strength)^9^. Specifically, in recent developments, the ability to microfabricate photolithographically patterned carbon features has further driven innovation in wider application of GC, one of the allotropes of carbon^1,2^. This technology involves pyrolyzing pre-patterned polymeric structure made from negative tone resists at high temperatures and inert environment in a tube furnace^1,2^.

Carbon is a compelling material for chronically implanted devices aiming to minimize tissue damage due to its chemical inertness, biocompatibility, good electrical properties, electrochemical stability, purely capacitive charge injection (no irreversible reactions and byproducts), and fast surface electrochemical kinetics^1–6^. We recently demonstrated neural probes with GC microelectrodes for electrocorticography (ECoG), intracortical recording and stimulation^3–5^. Brain stimulation applications require high performance microelectrode materials capable of sustaining hundreds of millions of cycles of electrical stimulations pulses (corresponding to implantations lasting 3–5 years) at clinically relevant charge densities without corrosion, tissue damage, or delamination of insulating and substrate layers. Meeting this requirement will enable the transition of neural probe technology from lab to clinical use, particularly with applications requiring long-term electrical stimulation, such as deep brain stimulation or spinal stimulation used for neuromodulation and induction of neuroplasticity^10^.

As a response to this need, we introduce here a new class of GC neural probes exclusively fabricated from a single homogeneous material of GC with no intermediate layers such as adhesion and conducting metal layers. This process removes any sources of potential mechanical or electrical discontinuities or failures at large cycles of electrical stimulations and extended period of *in-vivo* use. The new fabrication method presented here allows for a 2-step double-sided patterning, subsequent transfer to a polymeric supporting layer and final insulation of GC microelectrodes, interconnects and bump pads using a single material. In this new approach, therefore, there is no intermediate metal deposition process involved allowing–for the first time–all GC neural probes (aGC neural probes) made of a single homogenous material and supported on flexible polymeric substrate. The final aGC neural probes are housed in a flexible, thin-film polyimide substrate that leverage the superior electrochemical stability of GC material along with the improved biocompatibility of thin-film devices. To establish the robustness and superiority of the technology, extensive *in-vivo* and *in-vitro* mechanical, electrical, and electrochemical characterizations coupled with aggressive long-term stability and corrosion tests under electrical stimulations were carried out. This study demonstrates robustness and multi-modal recording capability for both neuronal electrical signals and neurotransmitter electrochemical signals. These probes are also capable of delivering balanced-charge pulsing over 3.5 billion cycles in extended accelerated aging process lasting more than 1000 hours. We demonstrate that these new probes have compelling advantages over existing technologies in almost all key metrics from mechanical and electrical properties to electrochemical kinetics, which is quite remarkable for probes fabricated from a single material without any surface coating.

## Materials and Methods

### Microfabrication

To demonstrate the technology, we microfabricated a 15-channel neural probe specifically designed for electrocorticography (ECoG) reading and a 4-channel penetrating probe designed and sized for neurotransmitter detection. The geometry of the ECoG device consisted of 300 μm diameter surface microelectrodes with 180 μm wide interconnects and a total width of 3.5 mm^2,3^. The neurotransmitter detection version had 4 penetrating microelectrodes of exposed area of 25 μm × 20 μm (500 μm^2^) spaced at 220 μm (see Supplementary Figures S1 and S2).

**Figure 1:**
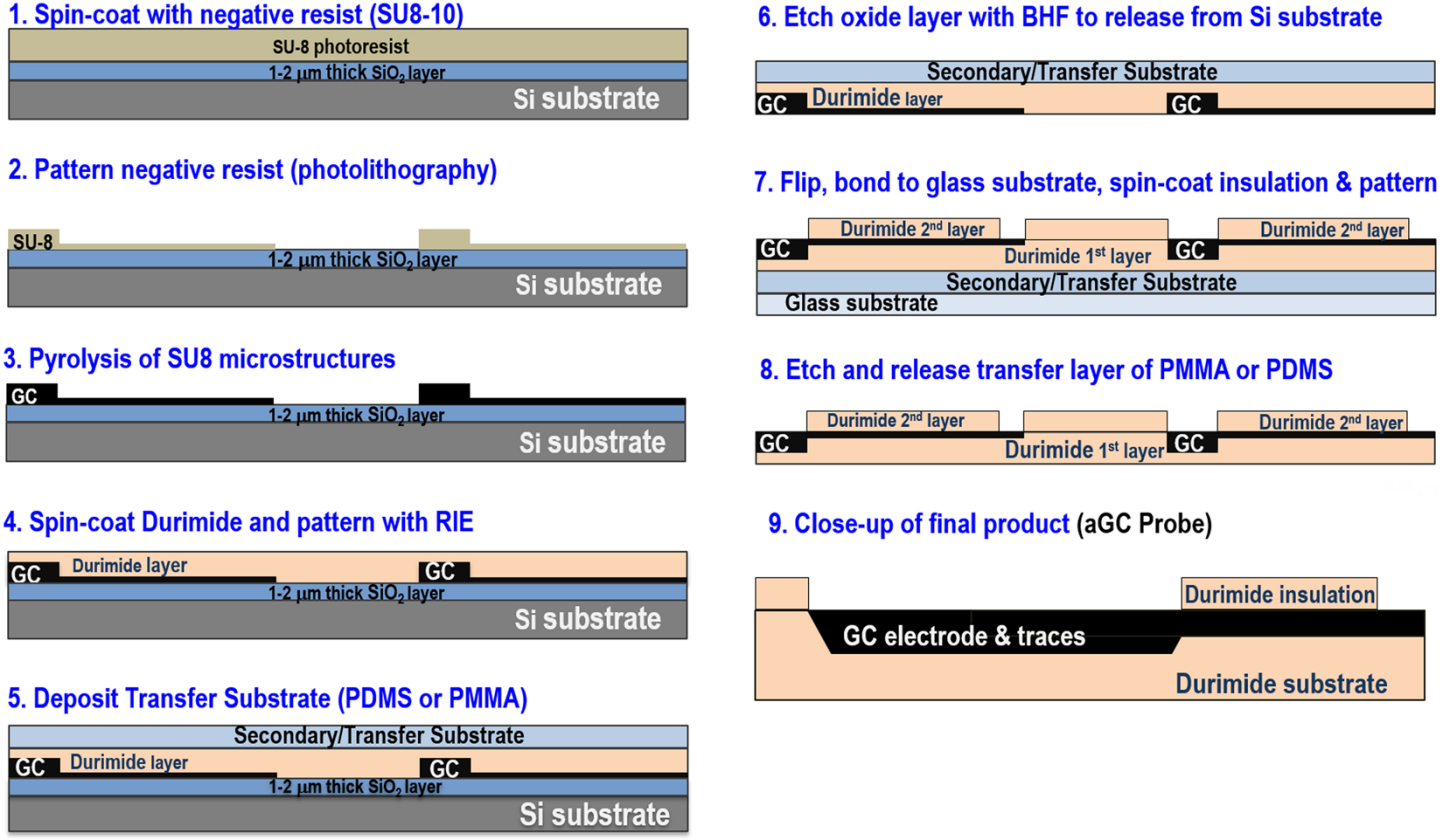
Lithography steps for microfabricating all glassy carbon (aGC) microstructures on a polymeric substrate. Two-step double-sided lithography patterning was used in addition to pyrolysis. Complete probes are referred as aGC probes whereas glassy carbon material is referred to simply as GC.

As shown in Fig. 1, the microfabrication process started with spin-coating 6 μm thick SU8–10 negative photoresist (Microchem, MA) and patterning it following conventional negative lithography process steps^1,2^. The patterned SU-8 microstructures were then pyrolyzed in a quartz furnace (MTI Corp., CA) under inert atmosphere of nitrogen gas with flow rate of 50 ml/min. For pyrolysis, the temperature was ramped to 1000 °C over a period of 6 hours and then held at this temperature for 90 minutes. Following this, the furnace was allowed to cool down to room temperature over a period of several hours.

Subsequently, the first base layer of ~8 μm thick non-photo sensitive polyimide (Durimide 115a) (Fujifilm, Japan) was spin-coated on top of GC microstructures at 2500 rpm for 45 seconds. This was then soft baked at 135 °C for 2.5 min and cooled down to room temperature. Patterning of Durimide was done through dry reactive ion etching (Technics Series 85 Reactive Ion Etcher, Pleasanton, CA) using Shipley positive resist (Microchem, MA) as a soft mask. The patterned Durimide layer was partially cured at 200 °C for 30 mins in N_2_ environment. A sacrificial transfer layer was then deposited as a secondary substrate on top of the Durimide layer. Polymers such as PMMA (Polymethyl methacrylate) or PDMS (Polydimethylsiloxane) could be used for this purpose. Here, we describe the use of PDMS (Dow Corning, USA) as one example. PDMS mixed with curing agent in 10:1 proportion was spin-coated at 300 rpm for 45 secs and then cured at 90 °C for 30 mins followed by BHF etching of oxide layer to release it from the silicon substrate. Subsequently, a glass substrate was bonded to the released structure to offer an additional temporary support for improved alignment. The wafer was then flipped and an insulation layer of Durimide spin-coated. After patterning this top insulation layer of Durimide, it was subsequently partially cured under N_2_. A final BHF etching removed the sacrificial layer and released all devices. The final aGC probes are shown in Supplementary Figures S1 (μECoG probe) and S2 (intracortical penetrating probe).

### Mechanical and Cross-Sectional Characterizations

The mechanical strength of the ensuing device was measured using Instron’s 1500HDX Universal Testing Machine (Instron, USA) where tensile load was applied till failure, and the resulting extension measured. Tensile test specimen probes with dimensions of 17 mm in length, 0.5 mm in width and 50 μm in thickness were used for the experimental study. Since the devices were too thin, attachment of strain gauge extensometers directly to the probes was not practical. Instead, linear displacement due to the crosshead motion of Instron was used to obtain displacement values. Thread-rolled C-clamps with clamping capacity of 0–25 mm were used to grip the probe. C-clamps provide greater precision for easy adjustment of position and prevent marring of the probe surface. The frame of C-clamps was then held between the jaws of upper and lower clamps of the Instron machine (ca. Supplementary Figure S4). The cross-head extension rate was set at 0.5 mm/min. The specimen was then loaded to failure at this rate and the load-deflection curve plotted. For comparison purposes, probes of similar geometry but consisting of 200 nm thick metal (Pt) microelectrodes and wire traces were microfabricated through standard metal lift-off process. Subsequently, the load-deflection curves and ultimate load carrying capacity of the thin-film metal probes were determined in a similar fashion. Young’s Modulus was determined by taking the mean slope of ten points in the elastic region of the stress-strain curve. The modulus of plain Durimide substrate with no metal or GC interconnects and microelectrodes was also determined separately to de-couple its effect from the composite structure. FEI Quanta 450 FEG SEM (ThermoScientific, OR) was used for scanning electron microscopy images. Cross sectioning was obtained through Focused Ion Beam (FIB) milling using a dual beam microscope system (Nova 600 NanoLab; FEI, Netherlands). This system is equipped with a field emission gun for scanning electron imaging and a focused ion beam of gallium ions for milling. The sectioning process was done in two steps: ion current milling (working at 30 kV and 7.7 pA) followed by a cleaning step of the section. Imaging was done at an acceleration voltage of 20 kV (0.4 nA of emission current).

### Electrical, Electrochemical, and Stability Characterizations

The electrochemical behavior of the microelectrodes was studied in Phosphate-Buffered Saline solution (PBS; 0.01 M, pH 7.4; Sigma Aldrich, USA). Cyclic voltammetry (CV) was used to quantify capacitive charging and microelectrode stability while electrochemical impedance spectroscopy (EIS) was used to determine the electrical properties of the probes over a large range of frequencies. For both CV and EIS tests, we used a potentiostat (Reference 600+, Gamry Instruments, USA) connected to a three-electrode electrochemical cell with a platinum wire as a counter electrode and a saturated Ag/AgCl reference electrode. During CV tests, the working electrode potential was swept between 1.3 V and −0.9 V (water window of GC^4^), maintaining a scan rate of 100 mV/s. The total charge storage capacity (CSC) was calculated from the time integral of a CV cycle. During EIS measurements, 10 mV RMS amplitude sine wave was superimposed on 0 V potential with frequency sweep from 0.1 to 10^5^ Hz. Equivalent circuit modeling of the EIS data was done through Gamry Echem Analyst Vn 7.05 software (Gamry Instruments, USA). For the accelerated aging test consisting of prolonged current stimulation patterns, we used a cathodic-first charge balanced bi-phasic current pulses with 450 µA amplitude, 400 μsec cathodic half-phase period and a frequency of 1 kHz in saline solution (0.01 M PBS) using a Gamry Instruments Virtual Front Panel (Gamry Instruments, PA, USA). This accelerated aging test corresponds to charge density of 0.25 mC/cm^2^ and was applied over a period of 1000 hours. Voltage transient responses during stimulation were acquired using the same potentiostat by simultaneously injecting stimulation current pulses and recording the corresponding voltage excursions between working and counter electrodes. In regular intervals, impedance was measured and the integrity of the electrodes checked. The buffer pH was measured regularly during accelerated aging test using pH/mv/ion benchtop meter (Jenco Electronics, TX).

Fast-Scan Cyclic Voltammetry (FSCV) was used to evaluate the electrodes’ dopamine detection performance with a WaveNeuro Potentiostat System (Pine Research, NC). For *in-vitro* dopamine calibration, the FSCV waveform was used with a pyramidal excursion from −0.5 V to +1.3 V potential and back to baseline with respect to the Ag/AgCl reference electrode at a scan rate of 400 V/s and 10 Hz frequency^11^. The duration of each scan was 9 ms (900 data points). Prior to the beginning of each experiment, the same voltage waveform was applied to the microelectrodes at 60 Hz for 1 hour for activating the carbon surface of the microelectrodes. Known concentrations of dopamine (10 nM–1 μM) were then infused over 5 seconds while changes in current were recorded for 20 seconds.

### *In-vivo* Tests

#### Micro-ECoG Implantation

All animal experiments were performed in accordance with the Association for Assessment and Accreditation of Laboratory Animal Care (AAALAC) Guide for the Care and Use of Laboratory Animals (8th Edition) and approved by the University of Washington Institutional Animal Care and Use Committee (IACUC) under protocol number 4265-01. Adult female Long-Evans rats (300 g) were used in this study and anesthesia was induced with a mixture of ketamine and xylazine. The animal was placed in a stereotaxic frame and a craniotomy made 1.5 mm rostral and 4.5 mm caudal to the bregma and 1 mm lateral and 7 mm lateral to the midline, targeting the left forelimb sensorimotor cortex. The microelectrode array was then placed on the dura and stabilized by gel foam dental cement. A reference wire was connected to a screw on the right parietal bone while a ground wire was connected to a screw on the occipital bone. Throughout the entire procedure, body temperature of the animal was maintained with a heating pad placed under it.

#### Electrophysiology Recording

Brain signal recording sessions were carried out under anesthesia, 4 days after array implantation. The implanted ECoG microarray was connected to a multichannel data acquisition system (Tucker-Davis Technologies, FL) through an active (unity-gain) head-stage. Sensory evoked potentials (SEP) were obtained by applying bipolar surface stimulation to the surface of skin of the right wrist of an anesthetized rat. Recorded data was digitized at 24.4 kHz and stored on a PC for further analysis. Subsequently, raw signals were bandpass filtered (1–450 Hz, 4th order Butterworth, zero-phase) and down-sampled to 1 kHz using Matlab software (Mathworks, Inc., MA). Low-frequency brain activity in evoked SEPs were eliminated by a high-pass filter (30 Hz, 4th order Butterworth, zero-phase). In each recording session, 15 single, bi-phasic, squared wave stimulation pulses (pulse width: 500 µs, pulse amplitude: 0.5 mA − 2 mA, 0.5 Hz) were applied to the surface electrodes and evoked response from 5 ms to 55 ms after stimulation onset were averaged to obtain average SEPs^12^. The extracted evoked response between 5 ms to 55 ms after stimulation onset was considered as an estimate for the signal, while the extracted spontaneous activity corresponding to 65 ms to 115 ms after stimulation onset was taken as an estimate for the background noise. Subsequently, SNR (signal-to-noise ratio) of ECoG electrodes placed in six different positions across forelimb sensory cortex were evaluated. The SNR value for each trial was defined as the ratio between the variance of selected signal (evoked response) and the variance of selected noise (spontaneous activity). The calculated SNR (mean ± standard deviation) of all channels was used as a useful indication of the quality of brain-signal recording. Furthermore, to show temporal-spectral representation of recorded data by GC microelectrodes, the spectrogram of each ECoG channel were computed by applying 256 ms Hanning window on each ECoG time-series. The corresponding short-time Fourier transform of each segment were then computed using 200 ms overlap.

## Results

In this section, we present the outcomes of the mechanical, electrical, and electrochemical characterizations.

### Optical and Cross-Sectional Characterizations

**Figure 2:**
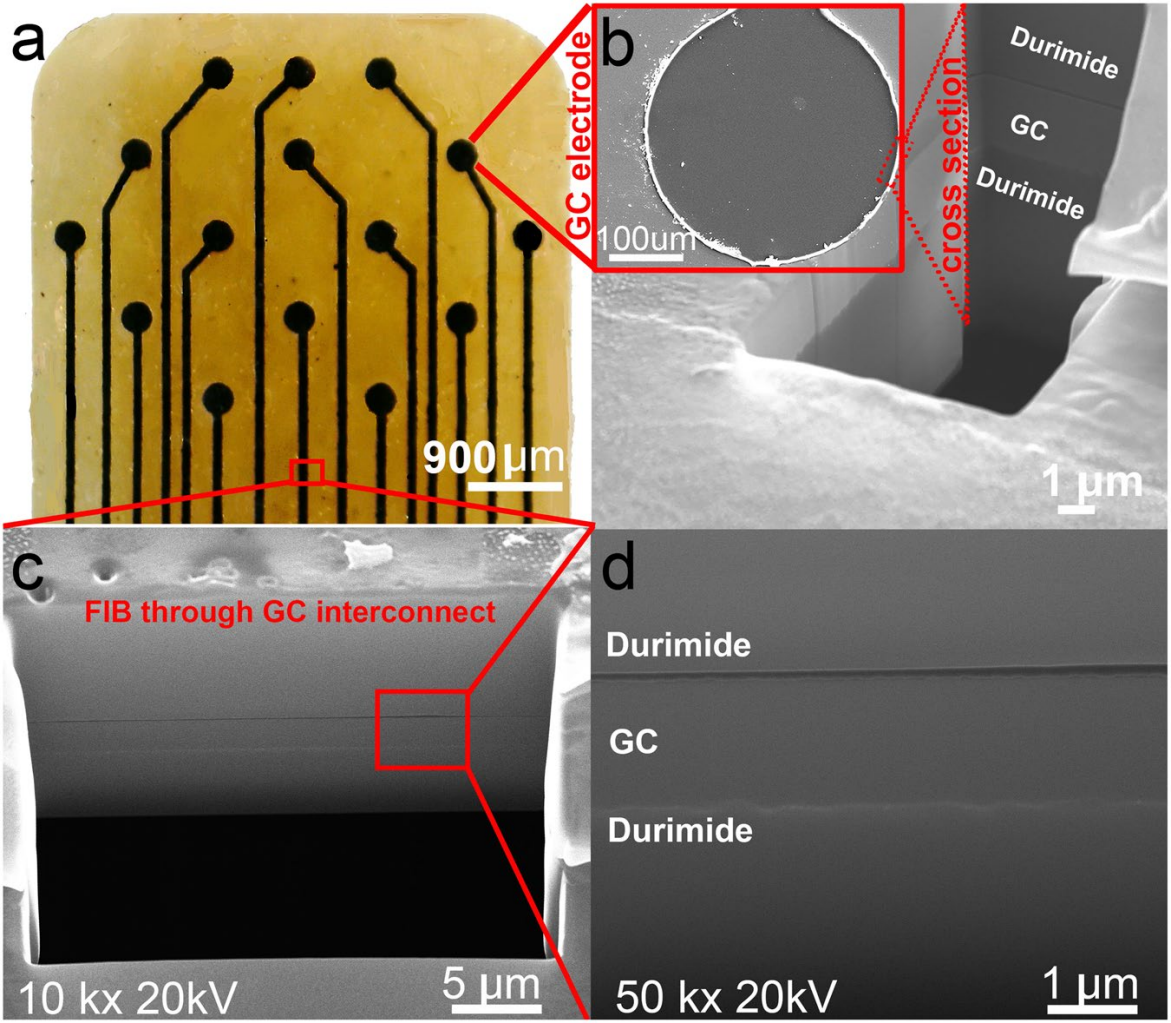
Details of aGC neural probe. (**a**) 15-channel aGC ECoG probe, (**b**) In inset: Magnified SEM image of a single microelectrode in the ECoG probe. Also, FIB cross-section of the GC microelectrode taken at the edge between GC and Durimide is shown revealing a seamless integration of the layers. (**c**) Expanded view of FIB cross-section taken at a GC wire trace leading to one of the microelectrodes, (**d**) High-resolution (50,000×) FIB image of a cross-section taken through a GC wire trace. Box in bottom of (**a**) shows the location where FIB through GC interconnect was taken. Note that there are no adhesion layers or metal layers in aGC probes.

Optical microscopy and SEM were used to visually investigate the structural integrity of the probes. As shown in Fig. 2a and Supplementary Figures S1–S3, the microelectrodes and interconnects (wire traces and bump pads) look sharp and well-defined after a two-stage, double-sided, pattern transfer procedure. Both the top and bottom insulation layers appear structurally robust. As can be seen in Fig. 2b,d, FIB characterizations of GC microelectrode/substrate and GC interconnect/substrate interfaces demonstrate that the top insulation and bottom substrate polymer layers have an almost seamless interface with GC suggesting a strong bond between GC and polymer layers. This smooth and seamless interface is an important requirement for a tight integration and composite action between GC and the polymer layers.

### Mechanical Characterizations

**Figure 3:**
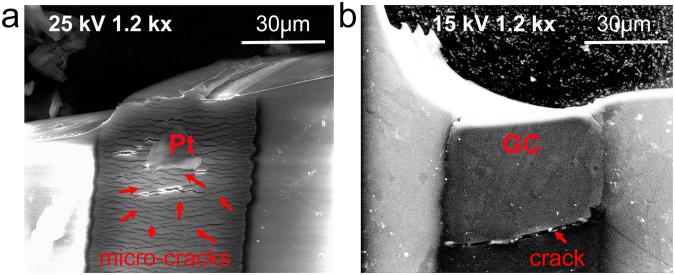
Details of fracture planes following mechanical testing of aGC and thin-film metal (Pt) probes. (**a**) SEM image highlighting failure plane in probes with metal interconnects where numerous micro-cracks were developed prior to and at time of failure. (**b**) SEM image of failure plane in aGC probes. Apart from the failure plane, a single crack is observed at failure while the rest of GC interconnects remain intact. This micro-crack extends through the width of the specimen consistent with cracks that form during failure of homogeneous materials.

Results from mechanical characterization are summarized in Fig. 3 showing failure planes at maximum load carrying capacity. Load-deflection curves under a tensile load are reported along with calculated modulus (Supplementary Figure S4). For comparison purposes, tensile load tests were carried out on probes with thin-film metal (Pt) microelectrodes and interconnects with similar geometry as their aGC counterparts. The load carrying capacity for both types of probes was directly obtained from the load-deflection curves and the resulting failure planes in transverse direction were further investigated using SEM. The aGC probes failed at a tensile load of ~12 N at 1 mm extension with clearly defined elastic and plastic regions observed in the load-deflection curves. On the other hand, probes microfabricated with thin-film metal (Pt) microelectrodes and traces failed at an ultimate tensile load of 5 N at 0.7 mm extension. For both thin-film metal and GC traces, the failure plane as shown in Fig. 3a,b passes through the traces with no jagged discontinuity, suggesting a strong bond between durimide and the respective metal or GC layer. The Young’s Modulus of the composite structure with GC microelectrodes and traces was calculated to be ~2.65 GPa whereas it was ~2.59 GPa for probes with Pt microelectrodes and traces. The modulus of aGC probes was slightly higher than that of plain Durimide (2.5 GPa) suggesting a strong composite action between GC and polyimide^3^. Optical observation revealed several micro-cracks in the thin-film metal traces near the failure plane (Fig. 3a), whereas only a single additional crack was seen in the otherwise intact GC trace (Fig. 3b), indicating different failure modes in the two types of probes. The extent of micro cracks in the thin-film metal electrodes suggests that they were progressively produced during loading resulting in earlier onset of electrical connection failure. For aGC probes, failure in the traces under tensile load seems to have occurred at higher load levels preceding plastic failure. Stiffness of aGC probes in compression was determined through the Euler buckling load: Pcr = π^2^EI/(KL)^2^, where E = Young’s modulus, I = area moment of inertia (500 μm width and 50 μm depth), K = effective length factor (usually 1) and L = length of probe. For the intracortical penetrating probes reported here, Pcr = 2.13 mN, which is substantially higher than ~1.5 mN force required to penetrate the cerebral cortex of a rat^13^.

### Electrical, Electrochemical and Stability Characterizations

**Figure 4:**
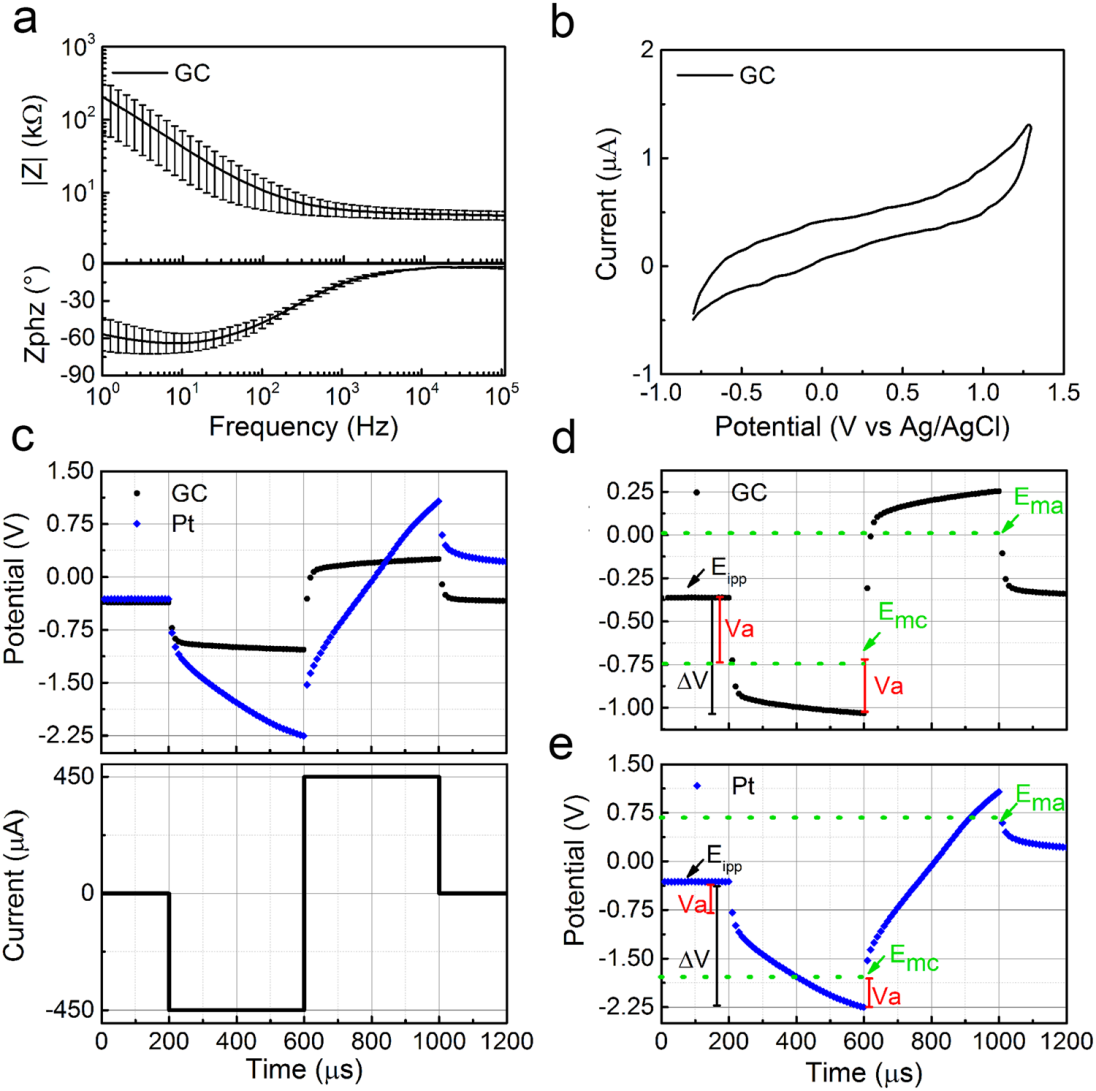
Electrochemical characterization of GC microelectrodes. (**a**) EIS plot (mean and standard deviation, n = 10), (**b**) representative cyclic voltammogram, (**c**) comparison of the voltage transient of GC versus Pt microelectrodes in response to a biphasic, symmetric 450 µA current pulse, (**d**) voltage transient response of GC microelectrode, (**e**) voltage transient response of Pt microelectrode. Both plots show the components that contribute to the voltage transients (i.e., V_a_, E_ipp_, E_mc_ and E_ma_). E_ipp_ is the inter-pulse potential. V_a_ is defined as the near-instantaneous voltage change at either the onset of the current pulse or immediately after the current pulse is terminated. It is associated with the Ohmic electrolyte resistance and overpotential terms^18^. Maximum positive polarization E_ma_ = V_max,pos_ − V_a_ whereas the maximum negative polarization E_mc_ = V_max,neg_ − V_a_. The values of E_mc_ and E_ma_ for GC and Pt are (−0.75 V, 0 V) and (−1.75 V, 0.75 V) respectively, indicating a lower overpotential and lower-threshold for electrochemical kinetics of GC microelectrodes.

Electrical characterization results are shown in Fig. 4. The impedance values at 10 Hz, 100 Hz and 1 kHz are (42.5 ± 27.6) kΩ, (10.7 ± 4.9) kΩ and (5.8 ± 1.2) kΩ, respectively (mean and standard deviation, n = 10). These indicate an excellent range for micro-ECoG recording applications. The corresponding EIS plots are reported in Fig. 4a. As the phase plot in Fig. 4a shows, impedances at frequencies higher than 4 kHz are dominated by almost exclusively a resistive component. Results from cyclic voltammetry of GC microelectrodes are summarized in Fig. 4b demonstrating an approximately rectangular shape, characteristic of microelectrodes exhibiting a predominantly double-layer capacitance. This is consistent with several other reports showing that this type of behavior is exhibited by only purely carbon materials, indicating that no faradaic reactions occur during the charging and discharging process^4,14–17^. CSC, calculated as the time-integral of an entire CV cycle between the water oxidation and reduction potential limits, is 61.4 ± 6.9 mC/cm^2^ (mean and standard deviation, n = 10).

This capacitive charge injection mechanism is also confirmed by the voltage transient shape (Fig. 4c) illustrating the comparison of voltage transients between GC and Pt microelectrodes with the same dimension of 300 µm diameter and comparable impedance. Traces are in response to a biphasic, symmetric current pulse of 450 µA amplitude and 400 μsec of half-phase period. Due to the capacitive charge injection mechanism of GC microelectrodes, the amplitude of voltage transient for the same current level applied to both types of probes is much less in GC microelectrodes, producing maximum negative voltage (V_max,neg_) of −1.03 V and maximum positive voltage (V_max,pos_) of 0.25 V. These correspond to E_mc_ (most negative polarization voltage) across electrode-electrolyte interface of −0.75 V and E_ma_ (most positive polarization voltage) close to 0 V^18^. These values are safely within the maximum potential limits of water reduction and oxidation for GC microelectrodes (i.e., −0.9 to 1.3 V; Fig. 4b). In contrast, for Pt microelectrodes, the corresponding V_max,neg_, E_mc_, V_max,pos_ and E_ma_ values are −2.25 V, −1.75 V, 1.07 V and 0.7 V, respectively. These values are substantially outside the maximum potentials corresponding to the water window of Pt at −0.6 V to 0.8 V. Note that the maximum negative potential excursion (E_mc_) is calculated by subtracting the access voltage (V_a_) associated with the Ohmic electrolyte resistance and overpotential terms from the maximum negative voltage in the transient (i.e., E_mc_ = V_max,neg_ − V_a_ and E_ma_ = V_max,pos_ − V_a_)^18^. V_a_ is defined as the near-instantaneous voltage change at either the onset of the current pulse or immediately after the current pulse is terminated^18^. In this condition, V_a_ is 0.36 V for GC and 0.48 V for Pt (33% higher for Pt), suggesting a substantially lower overpotential to be overcome in GC microelectrodes (Fig. 4c,d). Table 1 summarizes these results.

**Table 1:** Summary of key parameters in voltage transients for GC and Pt microelectrodes (continuous data in Fig. 4).

Material	ΔV	E_ipp_	V_a_	ΔE_p_	E_mc_	E_ma_	V_max,neg_	V_max,pos_
GC	0.72	−0.36	0.36	0.36	−0.75	0.05	−1.03	0.25
Pt	1.88	−0.31	0.48	1.4	−1.75	0.71	−2.25	1.07

E_mc_ = (V_max,neg_ − V_a_), E_ma_ = (V_max,pos_ − V_a_), and ΔE_p_ = ΔV − V_a_.

**Figure 5:**
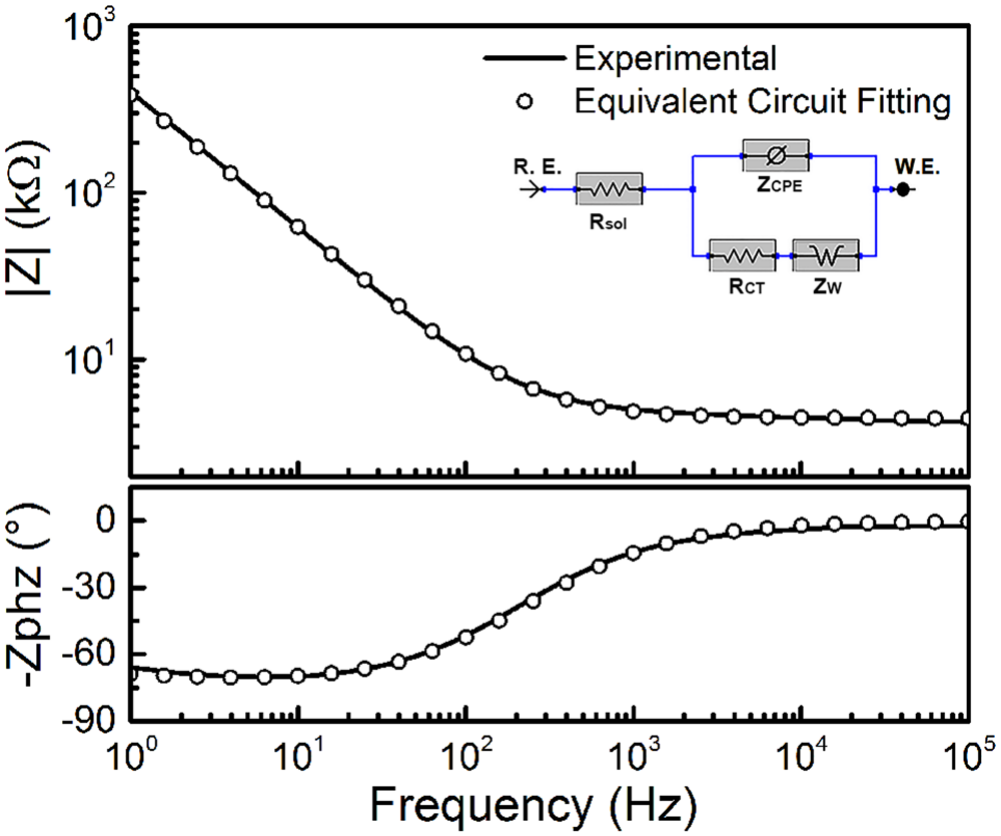
Equivalent circuit for aGC microelectrode based on modified Randles model. Representative EIS impedance spectra for experimental data (solid line) and equivalent circuit fitting (circles) used for curve fitting are shown. The optimal equivalent circuit model used (modified Randles circuit) is shown in inset. It is composed of electrolyte resistance (R_sol_) in series with a parallel combination of constant phase element (Z_CPE_), Warburg diffusion impedance (Z_W_), and charge transfer resistance (R_ct_). The EIS parameters obtained through fitting the experimental data to the model are: R_sol_ = 4.4 kΩ, Z_CPE_ with Y_0_ = 4.07 e^−11^ S*s^α^ and α = 0.87, Z_W_ = 2.74^−11^ S*s^1/2^, R_ct_ = 3.34 kΩ.

Further, to gain better understanding of the charge transfer properties of the microelectrodes, an equivalent circuit model was curve-fitted to the experimental EIS data. Representative EIS bode plots of the experimental data and curve-fitting of the most appropriate equivalent circuit model are reported in Fig. 5. The optimal model with the best fit is a modified Randles circuit that is composed of electrolyte resistance (R_sol_) in series with a parallel combination of charge transfer resistance (R_ct_), constant phase element (Z_CPE_), and Warburg impedance (Z_w_)^19–21^. The modified Randles equivalent circle selected here is consistent with results reported in the literature for capacitive microelectrodes^18–21^. In this model, the constant phase element Z_CPE_ is define as: Z_CPE_ = 1/(jω)^α^Y_o_ where Y_o_ represents the capacitance and α is a constant related to the angle of rotation in the complex plane compared to the purely capacitive behavior (Y_o_ = 1 for a pure capacitor)^19,20^. The corresponding parameters obtained by fitting the experimental data to the model are: R_sol_ = 4.4 kΩ, Z_CPE_ with Y_0_ = 4.07e^−11^ S*s^α^ and α = 0.87, Z_W_ = 2.74^−11^ S*s^1/2^, and R_ct_ = 3.34 kΩ.

**Figure 6:**
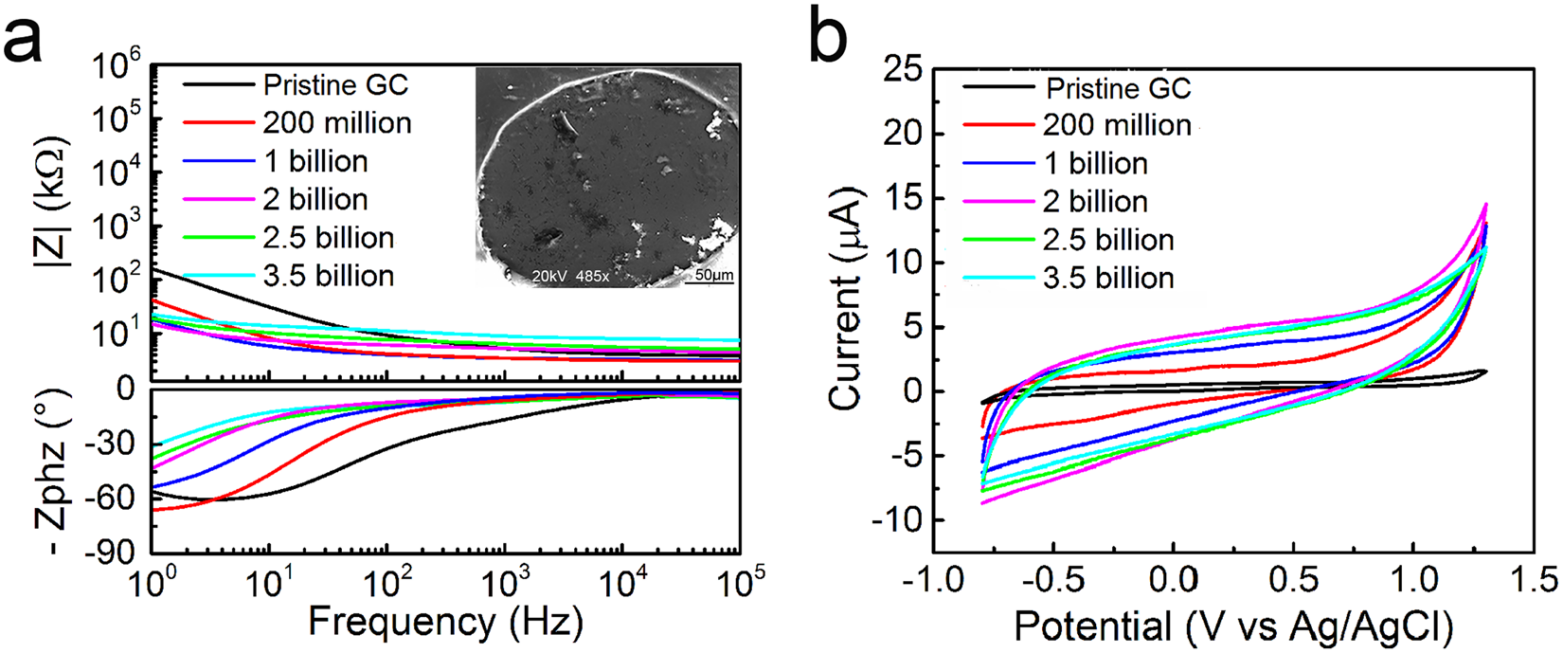
Effect of accelerated aging on microelectrodes through bi-phasic pulsing in PBS for 42 days (1000 hours). (**a**) Impedance spectra before and after 0.2, 1, 2, 2.5 and 3.5 billion cycles of stimulation pulses in PBS at 0.25 mC/cm^2^ charge density. Inset: SEM image of a GC microelectrode after 3.5 billion cycles of pulses. (**b**) Corresponding cyclic voltammograms over a range of stimulation pulses.

Results from accelerated aging test with cycles of electrical pulse applied over period of 1000 hours are reported in Fig. 6a,b. Figure 6 summarizes EIS plots, CV cycles and voltage transients of GC microelectrodes before and after 0.2, 1, 2, 2.5 and 3.5 billion cycles of electrical stimulation pulses. Prior to pulsing, for example, the impedance value at 1 kHz was ~5.2 kΩ. After 2, 2.5 and 3.5 billion cycles of pulses, the impedance at 1 kHz was measured to be 5.75, 6.3, 8.8 kΩ, respectively. In general, we observed a decrease in impedance at low frequency ranges with a shift of the low frequency pole and a corresponding increase in CSC. The CSC values increased from 45 mC/cm^2^ to 180 mC/cm^2^ after 0.2–3.5 billion cycles of pulsing. This increase in capacitance is predominantly due to continued surface activation that is very common in carbon microelectrodes^22^. The corresponding CV cycles are reported in Fig. 6b, exhibiting the expected rectangular shape of capacitive microelectrodes. Again, as CV diagrams demonstrate, there are no observable faradaic reactions occurring during charging and discharging processes.

As part of this accelerated aging test, pH value of ~7.55 was measured at several locations in the flow-cell, well within the buffer’s initial pH of 7.4. This is expected due to the buffering action of the PBS solution. Moreover, our findings are consistent with pH levels predicted by a transient FEA model in our previous work where pH near the microelectrode surface returned back to the initial conditions after each charge-balanced cycle^23^.

**Figure 7:**
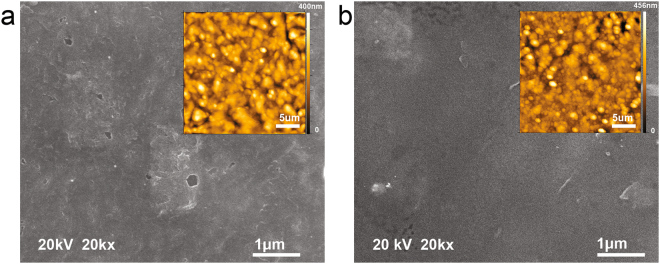
SEM and AFM image of microelectrode surface (**a**) before stimulation and (**b**) after 3.5 billion pulses of stimulation. The mean surface roughness was 43.55 ± 3.09 nm and 44.00 ± 6.69 nm (n = 4) before and after stimulation, respectively. *t-*test gave a nonsignificant difference (*P value* > *0.05*).

SEM and AFM inspections of GC microelectrodes demonstrate that the mechanical integrity of the microelectrode was still intact after 3.5 billion cycles of biphasic 0.25 mC/cm^2^ pulses in PBS solution over a period of 42 days (Fig. 7). SEM images confirm that the insulation and substrate polymeric layers remained intact and well attached to GC microelectrodes at the end of this accelerated aging under electrical stimulation. Further, as shown in Fig. 7, AFM images of the microelectrodes were taken at 25 μm × 25 μm area before and after aging to determine if there was any quantifiable and visible corrosion of the exposed microelectrodes. AFM morphology analysis indicated that, before stimulation, mean roughness of the microelectrode surface was 43.55 ± 3.09 nm (n = 4) while after stimulation, it was 44.00 ± 6.69 nm (n = 4). A corresponding *t-*test gave a nonsignificant difference (*P value* > *0.05*) between the mean roughness of the surfaces under the two conditions, confirming that there was no discernible corrosion after the aging process.

To test GC microelectrodes at near their charge injection limit, we applied a cathodic-first charge balanced bi-phasic current pulses with 5.1 mA amplitude, 400 μsec cathodic half-phase period and a frequency of 1 kHz in saline solution (0.01 M PBS) for 50 million cycles of pulses (3 mC/cm^2^). Voltage transients of a GC microelectrode in response to this biphasic pulse together with impedance spectra and the corresponding CV plots before and after 20, 40, 50 billion stimulation pulses are reported in Supplementary Figure S5, demonstrating that the microelectrode was able to sustain aggressive stimulation without significant changes in electrochemical performances.

### *In-Vivo* Electrophysiology Tests

**Figure 8:**
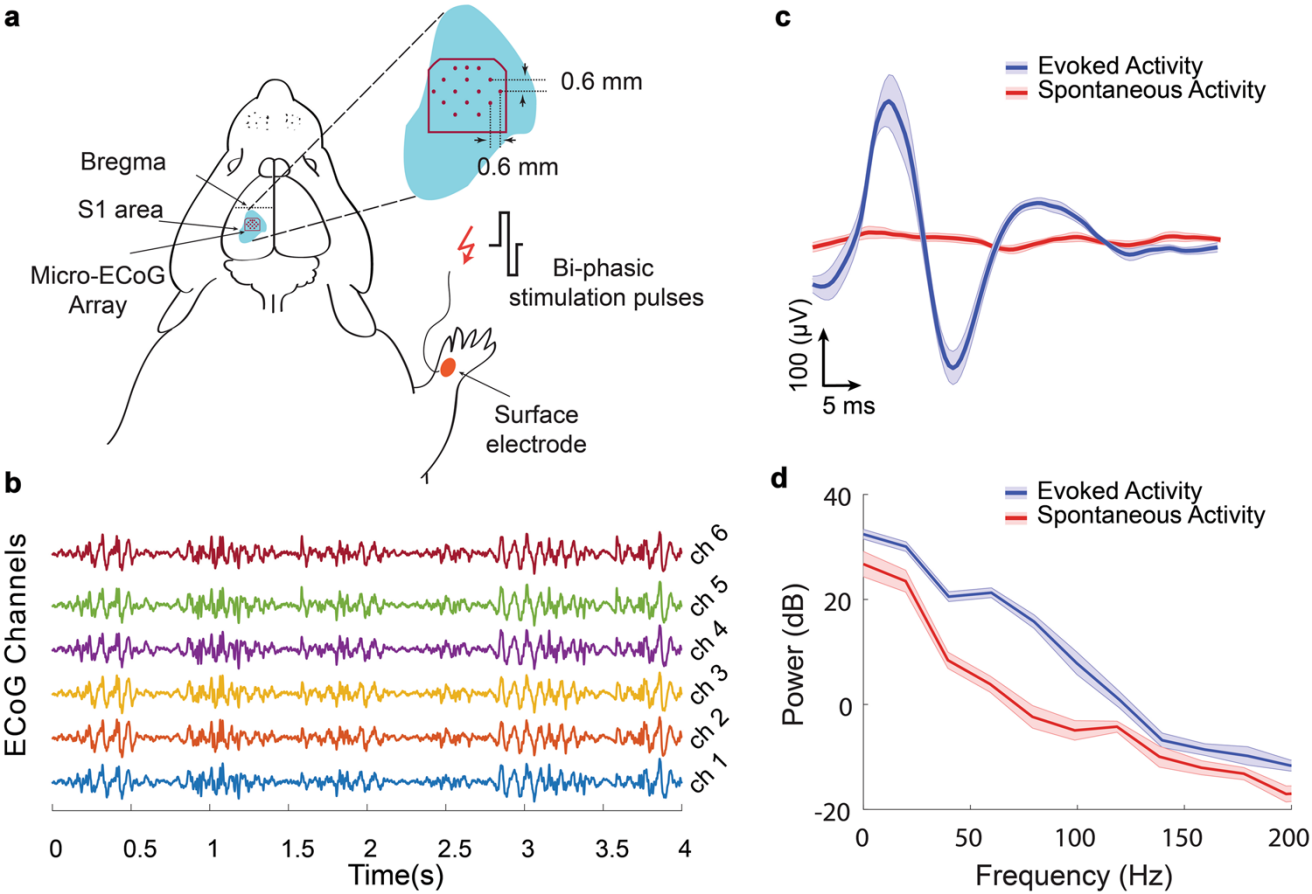
(**a**) Schematic figure showing implantation site of GC microelectrodes array on the left forelimb sensory cortex. Electrical stimulation was applied on the skin of the right wrist. (**b**) Example of raw ECoG data recorded using GC microelectrode arrays implanted on the forelimb area of rat sensory cortex. (**c**) Average-SEP response related 5–55 ms after surface electrical stimulation of hand skin with the average-spontaneous activity related to 65–115 ms after stimulation (**d**) the power spectrum (mean ± standard error) for sensory evoked response and spontaneous activities corresponding to the same signals shown in Fig. 8c.

SEPs caused by the bi-phasic stimulation pulses on the right wrist were recorded via the ECoG microelectrode array implanted on the left forelimb sensory cortex of an anesthetized rat. Figure 8a shows the craniotomy made targeting the left forelimb sensorimotor cortex where the probes were implanted. Plots of brain activities sampled from six channels of GC microelectrodes implanted in the sensory cortex four days after the implantation surgery are shown in Fig. 8b. To verify the quality of brain recording using GC microelectrodes, the average-SEP of different channels were calculated in response to the surface electrical stimulation of hand area. Figure 8c shows a typical average-SEP (mean ± standard error) of a single ECoG channel for 5 ms to 55 ms after stimulation onset, with average-spontaneous activity of the same ECoG channel for 65 ms to 115 ms after stimulation onset. The time frame between 5 ms to 55 ms was considered adequate to cover the entire SEP response, as all SEPs were observed in this time range. The large positive and negative response corresponding to 15 ms after stimulation onset demonstrates that the ECoG microelectrodes successfully recorded early response of sensory neurons in somatosensory cortex in the presence of background spontaneous activity. Figure 8d shows the power spectrum of the same six channels. As can be seen, all of the microelectrodes recorded brain activities in physiological spectral range without interference from non-physiological noise such as power line noise. Further, Supplementary Figure S6 shows that the spectrograms representing brain signals could be recorded across different frequency bands using GC microelectrodes. In Table 2, the mean (±standard deviation) SNR value (16 ± 6) and impedance of all ECoG channels are shown. Collectively, the *in-vivo* tests demonstrate that the GC ECoG microarray can successfully record multi-site, temporal and spectral brain information with high SNR.

**Table 2:** Average electrical recording SNR value and impedance of GC microelectrodes arrays implanted in the forelimb area of rat sensory cortex.

SNR (mean ± std)	*In-vivo* Impedance of Microelectrodes (kΩ) (mean ± std) (n = 8)	Impedance of Ground Electrode (kΩ)
16 ± 6	81 ± 16	<0.1

### Neurotransmitter (Dopamine) Detection

**Figure 9:**
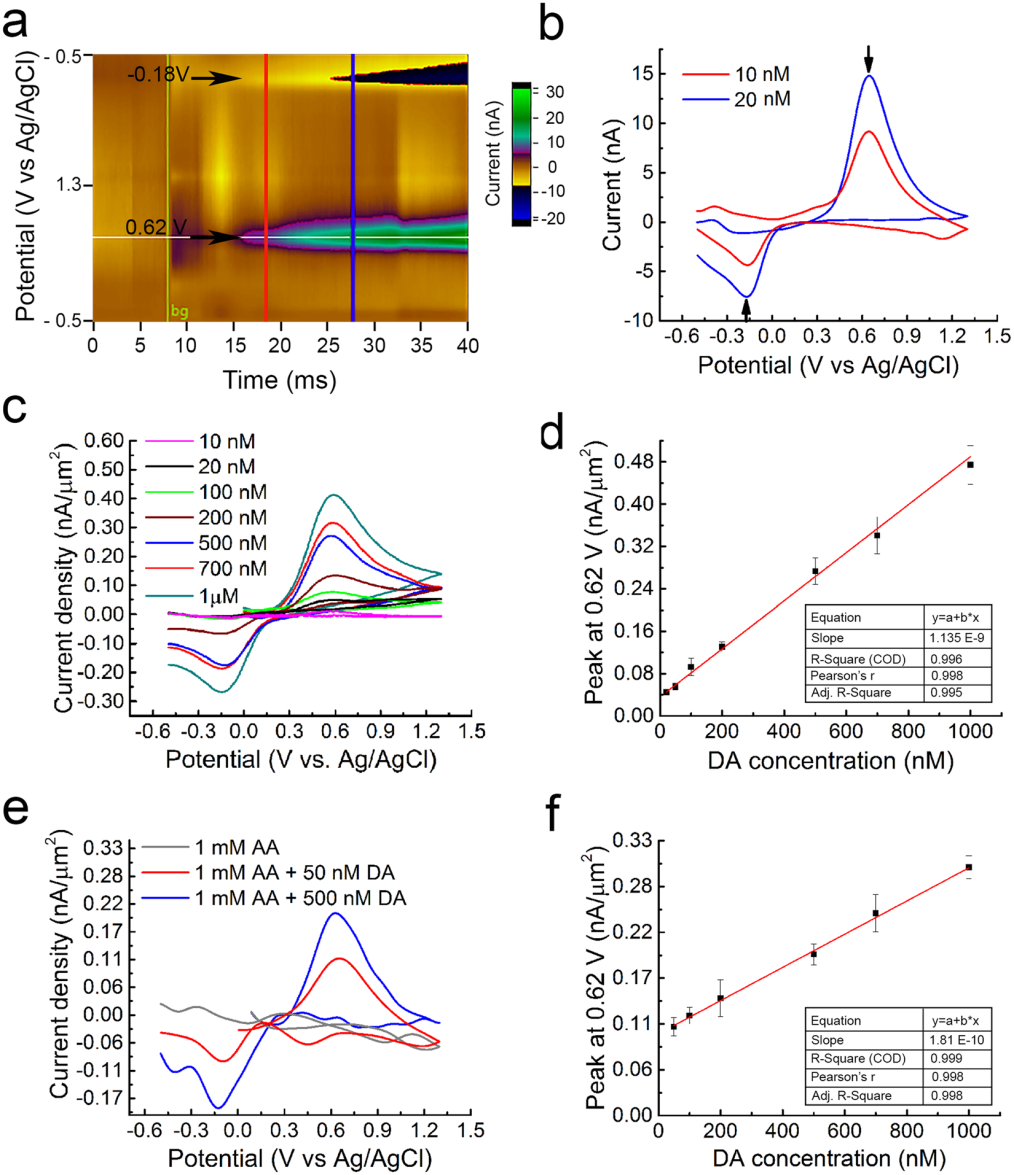
(**a**) Representative color plot showing changes in current following 10 nM dopamine injection in PBS at t = 15 secs and 20 nM injection at t = 25 secs (*in-vitro*), (**b**) corresponding background subtracted CV showing reduction (−0.18 V) and oxidation (0.62 V) peaks of DA. Dopamine signals were measured using a standard pyramidal FSCV waveform in which the applied voltage was ramped from the holding potential of −0.5 V to the switching potential of +1.3 V and then back to −0.5 V at 400 V/s. (**c**) Background subtracted CVs for 10,20,100,200,500,700 nM and 1 μM DA concentration in PBS, showing reduction (−0.18 V) and oxidation (0.62 V) peaks, (**d**) Calibration curve corresponding to conditions in Fig. 9c. (**e**) Background subtracted CVs showing reduction (−0.18 V) and oxidation (0.62 V) peaks of 50 and 500 nM of DA in presence of 1 mM AA in PBS; (**f**) Calibration curve of the oxidation (0.6 V) peak of 50, 100, 200, 500, 700 nM and 1 μM DA concentration in 1 mM AA in PBS.

Figure 9 summarizes the results obtained from a series of FSCV characterizations on a 500 μm^2^ net surface area penetrating GC microelectrode probes (Supplementary Figure S2). Figure 9a shows an example of dopamine signals detected using a standard pyramidal FSCV waveform in which the applied voltage was ramped from the holding potential of −0.5 V to the switching potential of +1.3 V and then back to −0.5 V at 400 V/s scan-rate. In particular, the color plots show changes in current following the injection of 10 nM of dopamine (DA) at t = 15 secs first and subsequently an additional 10 nM at t = 25 sec in the base PBS buffer. These changes in currents occurred at the known reduction and oxidation peaks of DA (i.e., −0.18 V and 0.62 V), as clearly shown in the background subtracted CVs for 10 nM and 20 nM DA given in Fig. 9b. Further, to determine a calibration curve over a wider range of concentrations, additional sets of FSCV were carried out at 10,20,50,100,200,500,700 nM and 1 μM concentration of dopamine in PBS. As shown in Fig. 9c, the background subtracted CVs corresponding to these range of concentrations demonstrate reduction and oxidation peaks at (−0.18 V) and (0.62 V). In addition, the calibration curve for these concentrations of DA varying from 10 nM to 1 μM follows a linear trend as reported in Fig. 9d. This low detection limit of dopamine at 10 nM represents one of the lowest reported limits in the literature.

Further, the capability of this aGC platform for selective detection of dopamine in the presence of Ascorbic Acid (AA) was also investigated. AA typically occurs in much higher concentration than that of DA (100–1000 times) and strongly interferes with DA detection^24^. Hence, the determination of DA with high selectivity and sensitivity in presence of AA is desirable for diagnostic applications. GC microelectrodes were able to detect 50 nM DA in the presence of 1 mM AA with high resolution. Background subtracted CVs showing reduction (−0.2 V) and oxidation (0.6 V) peaks of 50 nM and 500 nM of DA in presence of 1 mM AA in PBS are reported in Fig. 9e. The corresponding calibration curve shows a linear trend (Fig. 9f). This capability uniquely underscores an additional strong asset of GC microelectrodes in neurotransmitter detection through FSCV, a capability that is not matched by thin-film metal or metal oxide microelectrodes.

## Discussions

This study aims to overcome key barriers towards long-term clinical translation of neural probe technology. We demonstrate (i) long-term stimulation of electrodes at biologically relevant charge densities, (ii) ability to electrochemically sense neurotransmitters with the same platform as electrical signal recording, and hence potentially enable understanding of the interplay between the two signaling modalities, (iii) no corrosion of the electrode material and, (iv) no delamination of layer. The extensive characterizations reported here are targeted towards investigating these capabilities and establishing the suitability and superiority of GC microelectrodes towards advancing clinical translation of neural interfaces. Below, we review these in light of key results reported in the literature for other competing probe materials.

The mechanical and electrical integrity of the aGC probes, when exposed to aggressively large number of electrical stimulations cycles, is documented via SEM, AFM, FIB, EIS and CV characterizations. AFM and SEM images taken at several locations on the microelectrodes after accelerated aging show that there is no measurable and statistically significant difference on the surface roughness of the microelectrodes before and after aging. This strongly supports the observation that GC experiences no corrosion for voltage excursions applied within its wide electrochemical window of 2.4 volts^4^. Further, the maintenance of strong mechanical integrity of the probes after 1000 hours of accelerated aging under active electrical stimulation indicate that the attachment between GC microstructures and polyimide remains excellent. We had previously shown that the interface between GC and polyimide is dominated by strong hydrogen bonds through hydroxyl and carbonyl groups as well as covalent bonds through anhydride groups^25^.

It is important to closely examine if this exceptional performance in mechanical integrity is accompanied with equally superior electrical and electrochemical performance. For that, a closer examination of surface electrochemical kinetics in GC microelectrodes will provide useful parameters for assessing the performance of these microelectrodes under recording and electrical stimulation conditions. For example, CSC is typically an excellent indicator of the type of dominant current transduction in a microelectrode (i.e., faradaic Vs double-layer charging). In this study, we show that for GC microelectrodes made of a single and homogenous material with no coating of any kind, CSC is as high as 61.4 ± 6.9 mC/cm^2^. This value is slightly lower than that of carbon nanotubes (CNT) directly grown on metal microelectrodes (70.8 ± 1.1 mC/cm^2^)^14,15^, but much larger than porous graphene (50 mC/cm^2^)^17^. For further comparison, CSC for PEDOT-PSS coatings is 40.4 ± 4.8 mC/cm^2^ while it is 893.5 ± 137.8 mC/cm^2^ for PEDOT-PSS-CNT coatings and ~90 mC/cm^2^ for AIROF (Activated Iridium Oxide Film) microelectrodes^26–30^. All these microelectrode materials, however, utilize a coating on top of a thin-film metal layer. In the case of AIROF, it has an additional drawback of transferring charges through faradaic reactions that could result in microelectrode degradation and tissue damage. Therefore, our data support the interpretation that GC has the highest reported CSC for a single and homogenous probe material. In fact, if the surface of GC could be made porous through plasma etching or similar processes, the CSC value may further increase significantly.

Additional insight into electrode kinetics in GC microelectrodes can be obtained from voltage transients. The voltage transients in GC, in both negative and positive excursions, show a rapid and almost instantaneous convergence in the voltages suggesting a very fast electrode polarization. Pt microelectrodes, on the other hand, show a steep slope and slower polarization before V_max-neg_ or V_max-pos_ are reached. The voltage residuals at the end of each pulse cycle are also revealing in terms of the speed of charging and discharging. GC microelectrodes almost instantaneously converge back to starting potential whereas Pt still has significant voltage residual. In addition, electrode overpotential (η), which represents the deviation from the equilibrium potential (E − Eeq), is mainly driven by redox process and is a very good indicator of surface electrode kinetics^18,29–31^. The net voltage drop at the electrode is a sum of the Ohmic IR drop, electrode overpotential (drives redox at electrode/electrolyte interface), solution overpotential (due to transient charge distribution), and the shift in the equilibrium potential of the electrode (i.e., ΔV = IR + η_electrode_ + η_sol_ + ΔEo)^18^. Here, the same buffer is used for GC and Pt microelectrodes. Therefore, the differences in ΔV between the two materials are mostly due to electrode overpotential (η_electrode_), and to a lesser extent on solution overpotential (η_sol_). The overpotential of GC is significantly lower than that of Pt (0.38 Vs 0.48 V) suggesting that the voltage drop at the GC microelectrode surface due to overpotential accompanied by double layer charging and discharging is low (i.e., no reaction occurs). This is expected as overpotential is mainly influenced by faradaic reactions that are dominant in Pt microelectrodes.

With regard to accelerated aging under electrical stimulation, the charge density used in the experiments reported here (i.e., 0.25 mC/cm^2^) is consistent with the charge density used to evoke sensations in humans using millimeter-scale ECoG electrodes^32^. In the literature, there have been several reports of accelerated aging tests with and without electrical stimulation. With respect to passive (non-electrical) accelerated aging, thin-film Pt microelectrodes with adhesion promoters (i.e., polyimide-DLC-SiC-Pt) subjected to constant temperature of 37 °C for a period of 1 year^33^, silicon carbide microelectrodes immersed in a buffer heated to 96 °C for 1600 hours^34^, and PEDOT-coated microelectrodes immersed in buffer for 4 weeks have been reported^28^. These tests, however, were limited to either mechanical shear strength or current leakage of the probes. Electrical stimulation tests were reported for iridium microelectrodes with 2 billion cycles applied over ~167 hours (0.25 mC/cm^2^)^35^, porous graphene microelectrodes under 1 million biphasic current pulses^17^, GC with adhesion promoters (SiC and DLC) under 20 million pulses (0.2 mC/cm^2^)^36^, CVD-CNT-coated electrodes under 1 million pulses (1.6 mC/cm^2^)^15^, and PEDOT-coated electrodes subjected to 24-hour biphasic stimulation (3 mC/cm^2^)^29^. In general, the use of coatings is often a point of weakness, especially in cases where there is active electrical stimulation. A variety of materials such as titanium nitride, iridium oxide, PEDOT, and carbon nanotube composites have been adopted as coating layers^37–47^. However, these materials are prone to delamination during stimulation and long-term implantations, caused typically by poor adhesion between the coating material and the metal electrode^37,38,45^. Our current work is the first reported case where any neural probe, either with or without a coating, is subjected to 1000 hours and 3.5 billion cycles of active electrical current pulses. During these tests our probe exhibited no corrosion, delamination, or any sort of mechanical or electrical failure. Furthermore, on the same GC microelectrodes, we carried out an even more aggressive test at charge injection limit of 3 mC/cm^2^ with a cathodic-first charge balanced bi-phasic current pulses consisting of 5.1 mA amplitude, 400 μsec cathodic half-phase period and a frequency of 1 kHz in saline solution (0.01 M PBS) applied for 50 million pulses. Once again, GC microelectrodes survived this extreme test with no delamination of polymeric layers and no corrosion of electrodes. Further comparisons are given in Supplementary Table 1.

What drives this resistance to corrosion in GC microelectrodes? In the case of GC microelectrodes reported here, the ability to withstand such a high number of cycles of pulses can be explained by the fact that (i) GC is a homogeneous material with no crystalline structure; hence devoid of granular surfaces that could act as points of initiation of corrosive chemical reactions during stimulation^1,2^ and (ii) weaker metal adhesion layers have been eliminated. A previous study using AFM and SEM characterization demonstrated that surface roughness of GC is only a few nanometers, an order of magnitude smaller than typical thin-film metal electrodes^4,47^. In addition to this smoothness in surface, the current study has demonstrated that absence of a thin-film adhesion (Cr or Ti) and thin-film metal layer (Pt or Au) eliminates any potential Galvanic corrosion in probes^48^. This is a key distinguishing aspect of the technology presented here.

In addition, our demonstration of high-resolution real-time *in-vitro* neurotransmitter detection through background-subtracted FSCV using GC microelectrodes highlights the versatility and multi-modal (electrical and electrochemical) recording capabilities of the platform. Driven by its high spatiotemporal resolution and ability to detect sub-second fluctuations in neurotransmitter concentrations, FSCV has become a technique of choice for neurotransmitter detection. The 10 nM limit of detection (LOD) for dopamine reported here is one of the lowest ever reported in the literature. Given that biologically relevant dopamine concentration characteristic of living systems is within 26–40 nM^49^, such a low LOD represents significant progress in this area. There are several reports in the literature that document similar level of sensitivity. For example, CNT directly grown on metal microelectrodes (CNT-Nb) have shown dopamine detection limit of 11 ± 1 nM^50^, whereas SWCNT (Single Walled CNTs) on metal microelectrodes have shown LOD of 17 nM^51^. Further, for carbon nanofibers modified by PEDOT/graphene oxide coating, LOD of 85 nM was reported^52^ while laser-treated CNT yarn microelectrodes (CNTYMEs) have been shown to have LOD down to 13 ± 2 nM^53^. Further comparisons are given in Supplementary Table 2. As shown in the table, the GC LOD of 10 nM is superior to all other probes measured. Further, it is noteworthy that the majority of FSCV experiments reported in the literature were performed using carbon-fiber microelectrodes or single microelectrodes, allowing only single measurements. Glassy carbon microelectrodes, on the other hand, are suited for multi-site FSCV detection, similar to reported 3D carbon nanofiber microelectrode arrays (MEAs) fabricated through O_2_ plasma-assisted pyrolysis^54^, and pyrolyzed carbon MEAs using thin positive resists on non-biocompatible silicon substrates that were used for dopamine detection^55^.

An obvious question is what drives this high neurotransmitter sensitivity? GC surfaces–as reported here –- are ideal for detecting neurotransmitter electrochemical activity due to the intrinsic hydroxyl groups that form on their surfaces^25^. These hydroxyl groups are favorable for adsorption of cationic species such as dopamine whose amine side chain gets protonated at physiological pH^56^. Further, the strength of adsorption of dopamine at GC surface (and hence sensitivity) has been shown to correlate with increased heterogeneous electron transfer kinetics. This is an advantage of GC, as demonstrated in the voltage transients of Fig. 4c–e.

## Conclusions

In this study, we introduced the first use of all glassy carbon probe structure where microelectrodes, traces, and bump-pads are all fabricated from a single and homogeneous GC material. The probe experienced no measurable corrosion after a very aggressive accelerated aging test that involved 3.5 billion cycles of pulses. Through extensive characterizations, the versatility of this material, that may accelerate the translation of neuroprosthetic devices to clinical use, was demonstrated.

The following points highlight some of the key findings reported in this work:

1. The process introduced is compatible with MEMS and semi-conductor processes making the fabrication of these electrodes and their integration with CMOS a possibility.
2. GC microelectrodes have high SNR (>16) *in-vivo* recordings capability.
3. The accelerated aging introduced here is a record-setting 3.5 billion cycles of bi-phasic charge-balanced pulse train over period of 1000 hours. To the best of our knowledge, this is the first report on pulsing at this level and duration.
4. The corrosion rate in GC is very minimal and there was no difference between new and aggressively aged microelectrodes.
5. There was no failure at the GC and polyimide interface after 3.5 billion pulses and 1000 hours of immersion in a buffer. This suggests a strong mechanical robustness at the interface of microelectrodes and the supporting as well as insulating substrates aided by both hydrogen bonds (hydroxyl and carboxyl groups) and covalent bonds (anhydride groups).
6. GC microelectrodes have one of the highest reported CSC for single material non-coated neural probe (i.e., 61.4 ± 6.9 mC/cm^2^).
7. GC microelectrodes have one of the lowest limits of detection for dopamine at 10 nM. This is a significant finding.
8. The same probe materials are capable of stimulating, as well as recording both electrical and electrochemical signals (neurotransmitters). Therefore, GC microelectrodes offer a multi-modal platform that may accelerate investigation into the interplay between electrical and electrochemical signaling in the brain.

Collectively, our results demonstrate that GC may be an ideal microelectrode material for neuroprosthetics devices given its good impedance, excellent electrochemical response, high fidelity recordings, strong resistance to corrosion after billions of pulses of electrical stimulations, and ability to electrochemically detect neurotransmitters at one of the lowest concentrations reported. It is rare to find all these attributes in a single platform.

## Electronic supplementary material

Supplementary Figures and Tables
