# Supplementary material for: Ultra-Capacitive Carbon Neural Probe Allows Simultaneous Long-Term Electrical Stimulations and High-Resolution Neurotransmitter Detection
Source: Sci Rep. 2018 May 3;8:6958. doi: 10.1038/s41598-018-25198-x (PMC5934383; doi:10.1038/s41598-018-25198-x)
Supplement: Supplementary file 1 — Supplementary Figures and Tables [file 41598_2018_25198_MOESM1_ESM.docx]

**Ultra-Capacitive Carbon Neural Probe Allows Simultaneous Long-Term Electrical Stimulations and High-Resolution Neurotransmitter Detection**

**Surabhi Nimbalkar**^a,d,^**^§^, Elisa Castagnola**^a,d,^**^§^, Arvind Balasubramani**^a,d^**, Alice Scarpellini**^b^**, Soshi Samejima**^c,d^**, Abed Khorasani**^c,d^**, Adrien Boissenin**^c,d^**, Sanitta Thongpang**^c,d^**, Chet Moritz**^c,d^**, Sam Kassegne**^a,d,^**^^[[1]](#footnote-1)^^**

^a^ MEMS Research Lab, Department of Mechanical Engineering College of Engineering,

5500 Campanile Drive, San Diego State University, San Diego, CA, USA 92182

^b^ Department of Nanochemistry, Istituto Italiano di Tecnologia, Via Morego 30, 16163

Genoa, Italy.

^c^ University of Washington, Division of Physical Therapy Departments of Rehabilitation Medicine and Physiology and Biophysics, Seattle, WA

^d^ NSF-ERC Center for Sensorimotor Neural Engineering (CSNE)

**^§^** These authors contributed equally to this work.


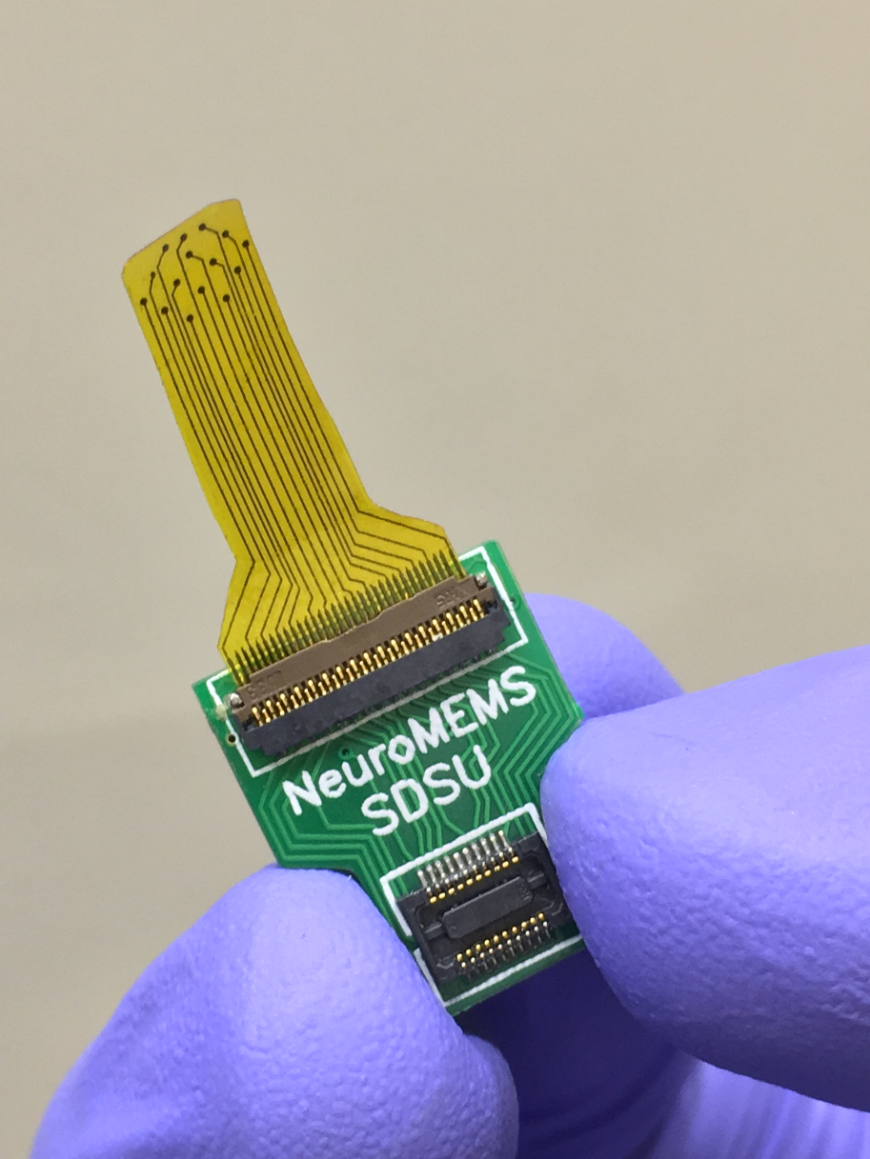


**Figure S1.** 15-channel aGC ECoG recording microelectrode array mounted on PCB with GC microelectrodes, GC traces and GC bump-pads microfabricated through 2-step double-sided patterning. The probe has microelectrodes with 300 μm diameter, 180 μm wide traces and a total width of 3.5 mm at the electrode locations. ZIF (Zero Insertion Force) connectors are used to connect the probes to PCB which is eventually connected to TDT (Tucker-Davis Technologies) multichannel data acquisition system via a DF30 connector (bottom).


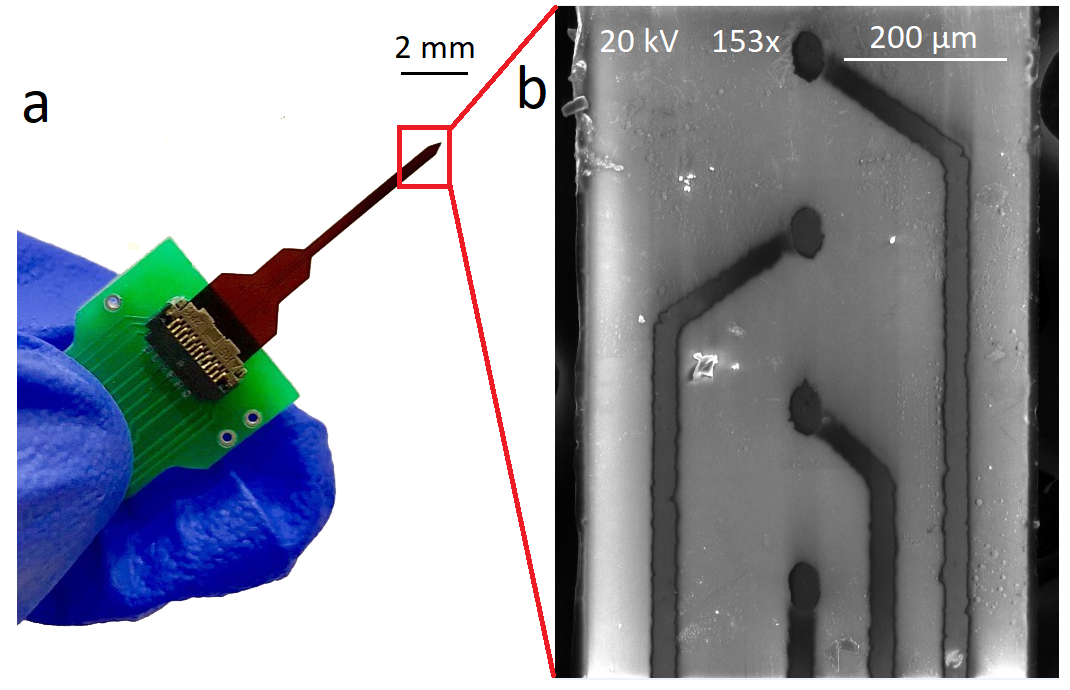


**Figure S2.** 4-channel aGC penetrating (intracortical) microelectrode array used for dopamine detection (a) Probe mounted on a PCB with ZIF connector (b) SEM image of aGC probe showing four GC microelectrodes with exposed area of 25 μm x 20 μm (500 μm^2^) spaced at 220 μm. Total width of the probe near the microelectrodes is 500 μm. Length of probes is 17 mm with width of 50 μm.


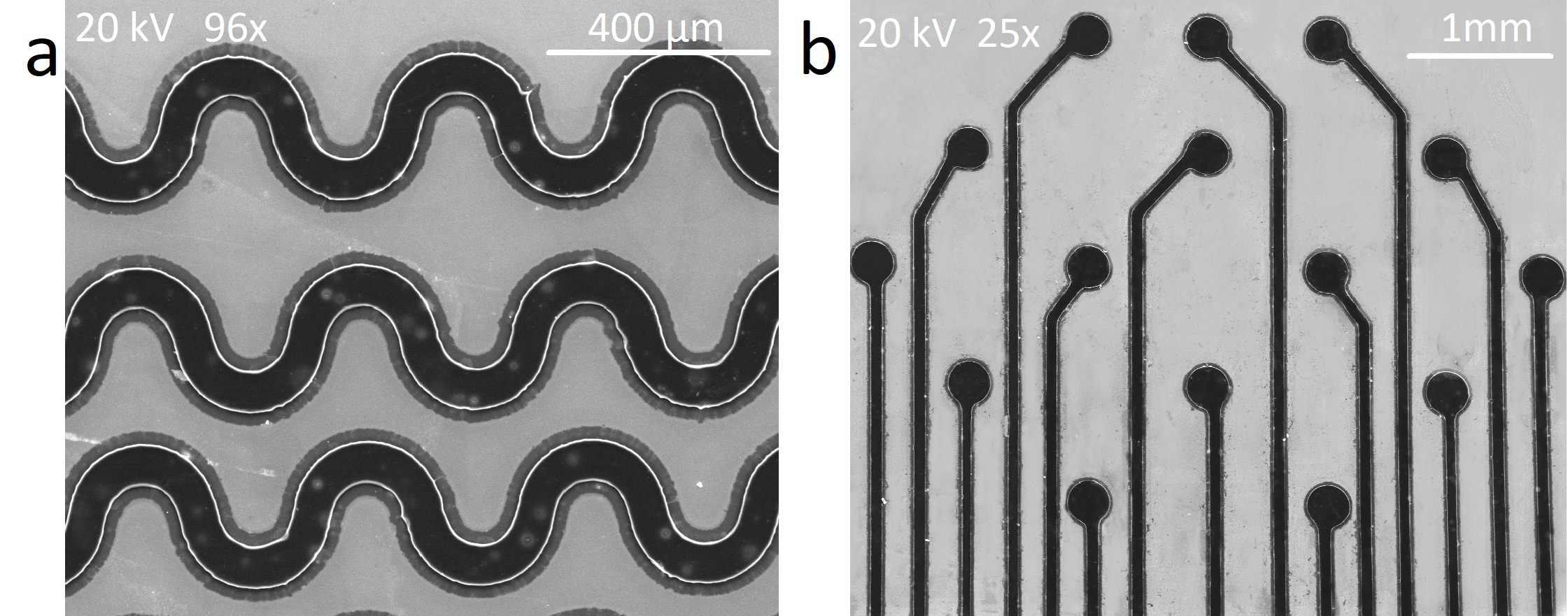


**Figure S3.** SEM Imaging after pyrolyzing SU8 photopolymer as precursor for GC traces and electrodes (a) Serpentine-shaped GC interconnects (b) Close-up view of GC microelectrodes (Ø = 300 μm) and GC interconnects.


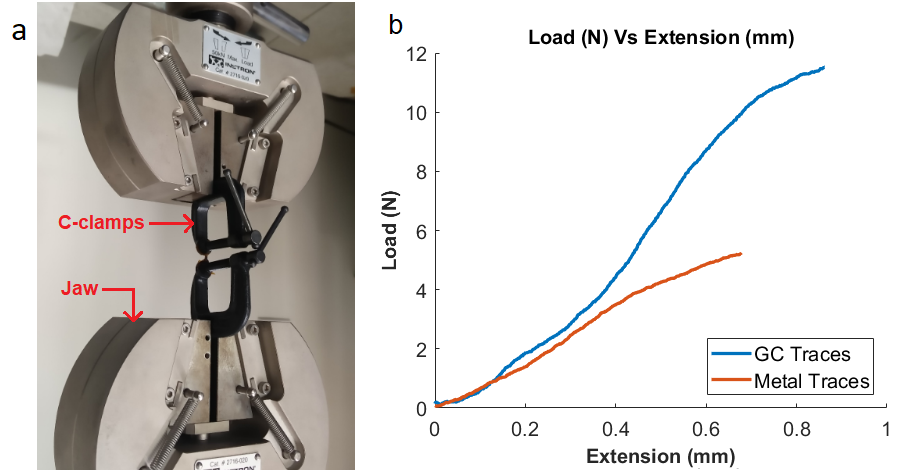


**Figure S4.** (a) Experimental setup of Instron Universal Testing Machine for axial tensile loading of aGC electrode probe (b) Comparison of load-deflection curve of aGC probes with that of thin-film metal probes of similar geometry. Tensile test specimen probes with 17 mm in length, 3 mm in width, and 50 μm in thickness were used.

**
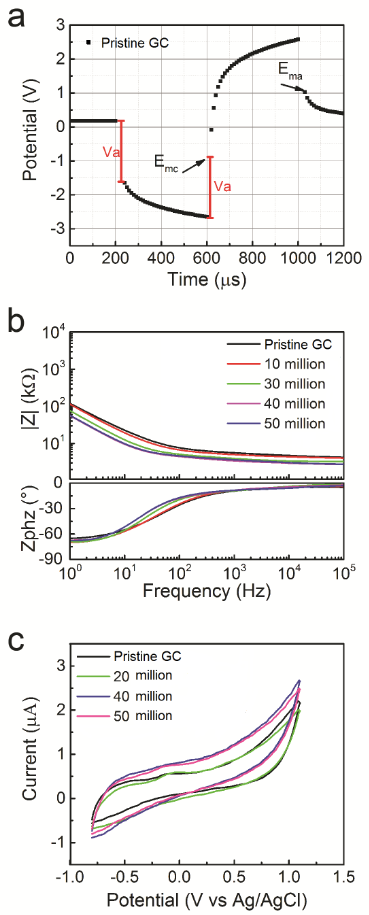
**

**Figure S5.** (a) Voltage transients of a GC microelectrodes in response to a 5.2 mA biphasic pulse corresponding to 3 mC/cm^2^ charge density (b) Impedance spectra before and after 20, 40, 50 billion cycles of 3 mC/cm^2^ stimulation pulses in PBS, and (C) Corresponding cyclic voltammograms. Qinj is calculated as the time integral of current in the loading phase normalized by the geometric area.

**
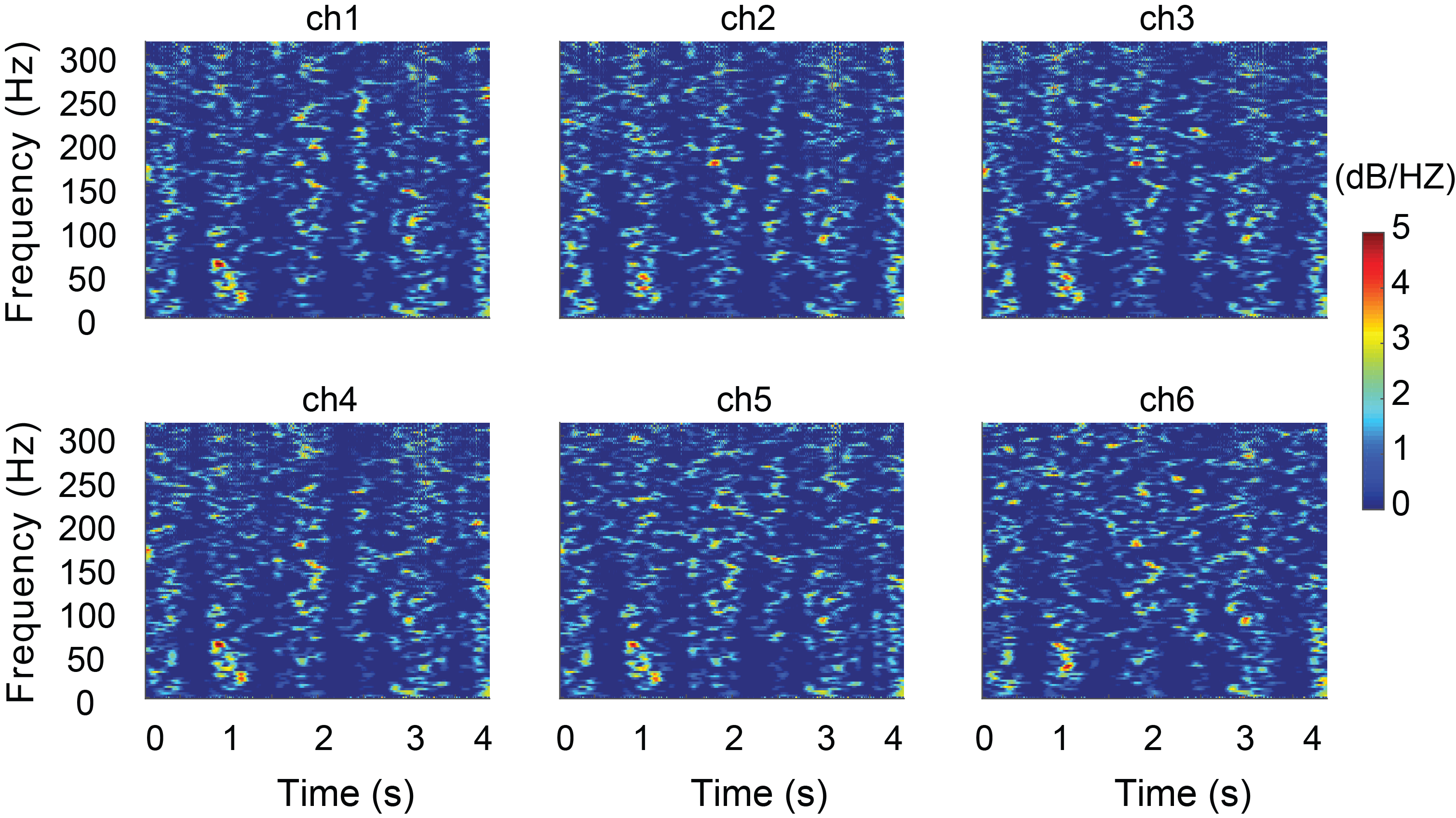
**

**Figure S6.** Spectrogram of six ECoG channels during SEP stimulation shown in Figure 8b. Short time Fourier Transform was applied to the 256 ms section of each ECoG time series and smoothed by 200 ms overlapped windows to obtain this temporal-spectral representation of ECoG data.

**Table 1: Summary of Charge Injection Limits (Qinj), CSC and Potential Limits of Microelectrode Materials Reported in Literature**

| **Material** | **Coating** | **CSC**  **(mC/cm^2^)** | **Qinj limit**  **(mC/cm^2^)** | **Potential limit**  **(V vs. Ag/AgCl)** | **Ref.** | **Mechanism** |
| --- | --- | --- | --- | --- | --- | --- |
| CNT pillars | no | -- | 1−1.6 | -1.5 - 1 | (1) | capacitive |
| CNT CVD | no | 70.8 ± 1.1  at 100 mV/s | 4 | -1 - 1.15 | (2) | capacitive |
| Porous Graphene | no | 50  at 100 mV/s | 3.2 | -1.3 - 0.8 | (3) | capacitive |
| PEDOT-CNT | yes | 40.4 ± 64.8,  158.7 ± 8.1,  540.7 ± 70.3  at 100 mV/s | 2.5 - 7 | -1 - 0.8 | (4-7) | pseudocapacitive |
| PPY-CNT | yes | 212.3 ± 23.9 | 7 | -0.9 - +0.6 | (2) | pseudocapacitive |
| PEDOT | yes | 75.6 ± 5.4,  893.5 ± 137.8  at 100mV/s | 2.3 - 15 | −0.8 - +0.6 | (6,8-10) | pseudocapacitive |
| IrOx | yes | 28.8 ± 0.3  at 50 mV/s | 0.9 - 3.3 | −0.8 - +0.6 | (8, 11-12) | faradaic |
| Pt | no | 0.5 ± 0.1,  8.6 ± 3.5 | 0.05 - 0.15 | -0.8 - +0.6 | (6, 8 ,9 13) | pseudocapacitive |
| GC  (Current) | no | 61.4 ± 6.9 | 3.0 | -0.9 - +1.3 |  | capacitive |

**Table 2. Summary of Detection Limits and Sensitivity to Dopamine Detection Reported in Literature**

| **Material** | **Detection Limit** | **Sensitivity at 1 μM DA** | **Ref** |
| --- | --- | --- | --- |
| CNT yarn microelectrodes | 25 ± 2 nM | -- | (14) |
| Laser treated CNT yarn microelectrodes | 13 ± 2 nM | -- | (14) |
| PEDOT/graphene oxide modified CF | 85 ± 9 nM | ca. 50 nA/μm^2^ | (15) |
| CF | 19 ± 4,  218 ± 20 nM | -- | (15,17,18) |
| Self-assembled SWCNT forests | 17 ± 3 nM  at 10 Hz,  65 ± 7 nM  at 90 Hz | 184 ± 19 pA/μm^2^ | (16) |
| CNT-Nb | 11± 3 nM | 197 ± 16 pA/μm^2^ | (17) |
| GC  (Current) | 10 nM | 450 ± 30 pA/μm^2^ |  |

**References**

1. K. Wang, H. A. Fishman, H. Dai, and J. S. Harri, *Neural Stimulation with a Carbon Nanotube Microelectrode Array*, Nano Lett., 2006, 6 (9), pp 2043–2048.
2. A. Ansaldo, E. Castagnola, et al., *Superior Electrochemical Performance of Carbon Nanotubes Directly Grown on Sharp Microelectrodes*, ACS Nano, 2011, 5 (3), pp 2206–2214.
3. Y. Lu, H. Lyu, A. G. Richardson, T. H. Lucas & D. Kuzum, *Flexible Neural Electrode Array Based-on Porous Graphene for Cortical Microstimulation and Sensing* Scientific Reports, 6:33526, DOI: 10.1038/srep33526:
4. X. Luo , C.L. Weaver, D.D. Zhou, R. Greenberg, X.T. Cui. *Highly stable carbon nanotube doped poly(3,4-ethylenedioxythiophene) for chronic neural stimulation*. Biomaterials.;32(24):5551-7, 2011, doi: 10.1016/j.biomaterials.2011.04.051.
5. E. Castagnola, L. Maiolo, E. Maggiolini, A. Minotti, M. Marrani, F. Maita, A. Pecora, G.N. Angotzi, A. Ansaldo, M. Boffini, L. Fadiga, G. Fortunato, D. Ricci, *PEDOT-CNT-Coated Low-Impedance, Ultra-Flexible, and Brain-Conformable Micro-ECoG Arrays*. IEEE Trans Neural Syst Rehabil Eng. ;23(3):342-50, 2015, doi: 10.1109/TNSRE.2014.2342880.
6. E. Castagnola, A. Ansaldo, E. Maggiolini,T. Ius, M. Skrap, D. Ricci, and L. Fadiga, *Smaller, softer, lower-impedance electrodes for human neuroprosthesis: a pragmatic approach*, Front. Neuroeng. 8, 2014 doi: 10.3389/fneng.2014.00008
7. E.Castagnola, E. Maggiolini, L. Ceseracciu, F. Ciarpella, E. Zucchini, S. De Faveri, L. Fadiga,and D. Ricci, *pHEMA Encapsulated PEDOT-PSS-CNT Microsphere Microelectrodes for Recording Single Unit Activity in the Brain*, Front Neurosci.; 10: 151, 2016. doi: 10.3389/fnins.2016.00151
8. S. F. Cogan, *Neural stimulation and recording electrodes*. Annu. Rev. Biomed. Eng. 10, 275–30910, 2008, 1146/annurev.bioeng.10.061807.160518
9. M. Vomero, E. Castagnola, F. Ciarpella, E. Maggiolini, N. Goshi, E. Zucchini, S. Carli, L. Fadiga, S. Kassegne and D. Ricci. *Highly Stable Glassy Carbon Interfaces for Long-Term Neural Stimulation and Low-Noise Recording of Brain Activity*, *Nature Sci. Rep.* **7**:40332 (2017) doi: 10.1038/srep40332.
10. S. Venkatraman, J. Hendricks, Z. A. King, A.J. Sereno, S. Richardson-Burns, D. Martin, and J. M. Carmena, *In Vitro and In Vivo Evaluation of PEDOT Microelectrodes for Neural Stimulation and Recording*, IEEE Trans Neural Syst Rehabil Eng., VOL. 19, NO. 3, JUNE 2011 307.
11. S. F. Cogan, P.R. Troyk, J. Ehrlich, T.D.Plante, D.E. Detlefsen, *Potential-biased, asymmetric waveforms for charge-injection with activated iridium oxide (AIROF) neural stimulation electrodes*. IEEE Trans. Biomed. Eng. 53, 327–33210, 2006.
12. S. J. Wilks, S. M. Richardson-Burns, J. L. Hendricks, D.C. Martin, and K. J. Otto, *Poly(3,4-ethylenedioxythiophene) as a Micro-Neural Interface Material for Electrostimulation*, Frontiers in Neuroengineering., 2: 7., 2009; doi: 10.3389/neuro.16.007.
13. L. Bullara, D. McCreery, T. Yuen, W. Agnew, A microelectrode for delivery of defined charge densities. Journal of Neuroscience Methods 9, 15–21, 1983.
14. C. Yang, E. Trikantzopoulos, M. D. Nguyen, C. B. Jacobs, Y. Wang, M. Mahjouri-Samani, I. N. Ivanov, and B. J. Venton, *Laser Treated Carbon Nanotube Yarn Microelectrodes for Rapid and Sensitive Detection of Dopamine in Vivo*, DOI: 10.1021/acssensors.6b00021 ACS Sens. 2016, 1, 508−515
15. I.M. Taylor, E.M. Robbins, K.A. Catt, P.A. Cody, C.L. Happe, X.T. Cui. *Enhanced dopamine detection sensitivity by PEDOT/graphene oxide coating on in vivo carbon fiber electrodes, Biosens Bioelectron*., 89 (Pt 1):400-410, 2017. doi: 10.1016/j.bios.2016.05.084.
16. N. Xiao and B. J. Venton, *Rapid, sensitive detection of neurotransmitters at microelectrodes modified with self-assembled SWCNT forests*, Anal Chem. 84(18): 7816–7822, 2012,.doi: 10.1021/ac301445w
17. C.Yang, C. B. Jacobs, M. D. Nguyen, M. Ganesana, A. G. Zestos, I. N. Ivanov, A. A. Puretzky, C. M. Rouleau, D. B. Geohegan, and B. J. Venton, *Carbon Nanotubes Grown on Metal Microelectrodes for the Detection of Dopamine*, Anal. Chem., 88, 645−652, 2016, DOI: 10.1021/acs.analchem.5b01257
18. W. Yi, Y. Yang, P. Hashemi, M. M.-C,Cheng, *3D carbon nanofiber microelectrode arrays fabricated by plasma-assisted pyrolysis to enhance sensitivity and stability of real-time dopamine detection*, Biomed Microdevices 18: 112, 2016, DOI 10.1007/s10544-016-0136-1.

1. Address correspondences to Sam Kassegne • Professor of Mechanical Engineering, MEMS Research Lab, Department of Mechanical Engineering, College of Engineering, San Diego State University, 5500 Campanile Drive, CA 92182-1323. E-mail: [kassegne@mail.sdsu.edu](mailto:kassegne@mail.sdsu.edu) • Tel: (760) 402-7162. [↑](#footnote-ref-1)
